# Supplementary material for: Evidence into practice: protocol for a new mixed-methods approach to explore the relationship between trials evidence and clinical practice through systematic identification and analysis of articles citing randomised controlled trials
Source: BMJ Open. 2018 Nov 8;8(11):e023215. doi: 10.1136/bmjopen-2018-023215 (PMC6231588; doi:10.1136/bmjopen-2018-023215)
Supplement: Supplementary file 1 [file bmjopen-2018-023215supp001.pdf]

SUPPLEMENTARY MATERIAL

Supplementary table 1. Document types indexed by Web of Science and Scopus.

Web of Science	Scopus
Article	Article
Abstract of published item	Abstract report
Art exhibit review	Article in press
Bibliography	Book
Biographical-item	Business article
Book	Book chapter
Book chapter	Conference paper
Book review	Conference review
Chronology	Editorial
Correction	Erratum
Correction, addition	Letter
Dance performance review	Note
Data paper	Press release
Database review	Review
Discussion	Short survey
Editorial material	
Excerpt	
Fiction, creative prose	
Film review	
Hardware review	
Item about an individual	
Letter	
Meeting abstract	
Meeting summary	
Music performance review	
Music score	
Music score review	
News item	
Note	
Poetry	
Proceedings paper	
Record review	
Reprint	
Retracted publication	
Retraction	
Review	
Script	
Software review	
TV review, radio review	
TV review, radio review video	
Theatre review	
