# Supplementary material for: Evidence into practice: protocol for a new mixed-methods approach to explore the relationship between trials evidence and clinical practice through systematic identification and analysis of articles citing randomised controlled trials
Source: BMJ Open. 2018 Nov 8;8(11):e023215. doi: 10.1136/bmjopen-2018-023215 (PMC6231588; doi:10.1136/bmjopen-2018-023215)
Supplement: Author's manuscript [file bmjopen-2018-023215.draft_revisions.pdf]

BMJ Open is committed to open peer review. As part of this commitment we make the peer review history of every article we publish publicly available.

When an article is published we post the peer reviewers' comments and the authors' responses online. We also post the versions of the paper that were used during peer review. These are the versions that the peer review comments apply to.

The versions of the paper that follow are the versions that were submitted during the peer review process. They are not the versions of record or the final published versions. They should not be cited or distributed as the published version of this manuscript.

BMJ Open is an open access journal and the full, final, typeset and author-corrected version of record of the manuscript is available on our site with no access controls, subscription charges or pay-per-view fees (http://bmjopen.bmj.com).

If you have any questions on BMJ Open's open peer review process please email
info.bmjopen@bmj.com

BMJ Open

Evidence into practice: protocol for a new mixed methods approach to explore the relationship between trials evidence and clinical practice through systematic identification and analysis of articles citing randomised controlled trials.

Journal:	*BMJ Open*
Manuscript ID	bmjopen-2018-023215
Article Type:	Protocol
Date Submitted by the Author:	02-Apr-2018
Complete List of Authors:	Byrne, Benjamin; University of Bristol, Centre for Surgical Research, Population Health Sciences<br>Rooshenas, Leila; University of Bristol, Centre for Surgical Research, Population Health Sciences<br>Lambert, Helen; University of Bristol, Population Health Sciences<br>Blazeby, Jane; University of Bristol, Centre for Surgical Research, Population Health Sciences
Keywords:	QUALITATIVE RESEARCH, STATISTICS & RESEARCH METHODS, SURGERY, Clinical trials < THERAPEUTICS

Evidence into practice: protocol for a new mixed methods approach to explore the relationship between trials evidence and clinical practice through systematic identification and analysis of articles citing randomised controlled trials.

B E Byrne[1], L Rooshenas[1], H Lambert[2], J M Blazeby[1]

[1]Centre for Surgical Research, Population Health Sciences, Bristol Medical School, University of Bristol, Bristol, UK.

[2]Population Health Sciences, Bristol Medical School, University of Bristol, Bristol, UK.

Correspondence: B E Byrne

Address: Centre for Surgical Research, Population Health Sciences, Bristol Medical School, University of Bristol, Canynge Hall, 39 Whatley Road, Clifton, Bristol BS8 2PS, UK.

Email: benbyrne@doctors.org.uk.

Telephone: 0117 928 7279

For peer review only

For peer review only - http://bmjopen.bmj.com/site/about/guidelines.xhtml

ABSTRACT

Introduction: Randomised controlled trials (RCTs) provide high-quality evidence to inform practice. However, much routine care is not based upon available RCT evidence. Understanding this disconnect may improve trial design, reporting and implementation. Published literature citing, critiquing and commenting on RCTs may yield relevant insights. This protocol presents a new approach examining how researchers understand, contextualise and use evidence from RCTs, through analysis of citing articles. Surgical case studies will illustrate its ability to identify wide ranging factors influencing application of trials evidence.

Methods and analysis: In-depth study of published literature will explore written clinical and academic responses to RCTs. After purposefully selecting individual RCTs, Web of Science and Scopus searches will systematically identify citing articles. Editorial, discussion and letter publication types will be included. These are considered most likely to provide critiques and opinions about the index RCTs. Original articles and reviews will be excluded. Information on clinical specialty, RCT design, outcomes and bibliographic data will be collected for the RCTs and citing articles. Citing articles will be thematically analysed using the constant comparison approach to explore author understanding, contextualisation and relationship to clinical practice for the index trial. Coding will include generic issues relevant to all RCTs and features specific to surgery. Index trial quality (internal and external validity and context) will be examined using validated tools. Results will be combined to create a broad overview of the understanding and use of RCT evidence.

Ethics and dissemination: This study involves secondary use of existing articles and does not require ethical approval. Pilot work will establish its feasibility and inform progression to larger-scale utilisation across a broad range of RCTs. Findings will be published in a peer reviewed journal and

For peer review only - http://bmjopen.bmj.com/site/about/guidelines.xhtml

For peer review only

presented at surgical and methodological conferences. Results will guide future work on trial design to optimise implementation of results.

For peer review only

For peer review only - http://bmjopen.bmj.com/site/about/guidelines.xhtml

ARTICLE SUMMARY

Strengths and limitations of this study

- This new method will use published response to RCTs to identify how clinicians and academics interpret and use evidence from randomised trials.
- The emerging data will identify areas that need attention to improve translation of RCT results into practice.
- Rigorous qualitative analysis combined with structured data extraction on the quality and reporting of included RCTs will be used to gain in-depth understanding of the relevant issues.
- This method may have broad applicability across all randomised trials.
- The study findings may be limited by the focus on included and published articles.

For peer review only

For peer review only - http://bmjopen.bmj.com/site/about/guidelines.xhtml

INTRODUCTION

Since the early description of evidence-based medicine(1), it has been recognised that high quality research is needed to improve clinical practice and patient outcomes. This typically includes well-designed and conducted randomised controlled trials (RCTs) and systematic reviews summarising the results of multiple trials in one field(2). Such research is used to inform national guidelines which aim to influence and set standards for clinical practice.

Research across a number of areas, including surgery, has shown that day-to-day care is often not delivered in accordance with national guidelines and best evidence(3–6). There are many reasons for lack of translation of knowledge from RCTs into practice. Trials may be compromised by problems with their design and conduct, limiting the credibility of the associated results and / or the applicability of the trial in other settings. Internal validity can be compromised by well-known sources of bias that may be assessed with validated tools, such as the Risk of Bias Tool(9). Trial design and reporting may also compromise external validity, limiting the application of trial findings more broadly(10,11). In addition, complex interventions, such as surgery, may be influenced by a wide range of factors, such as surgeon or institution caseload, teamwork, care pathways and organisational culture, which are generally referred to as 'context'(12–14). The exact mechanisms by which such contextual elements could affect the results of care have not been defined, but they may include technical skills, decision-making, professional status that may influence quality of post-surgical care and communication. These may have clear relevance to both the efficacy and implementation of an intervention.

Other factors beyond the control or influence of trials methodologists are also important in determining whether evidence from RCTs is implemented in practice. Evidence from an individual RCT regarding a particular intervention must be considered alongside a range of other evidence

For peer review only - http://bmjopen.bmj.com/site/about/guidelines.xhtml

types, including RCTs concerning the same or similar interventions. Clinicians, many of whom have not been involved in clinical trials, may lack familiarity with trial design; even those involved in trial recruitment may have a poor grasp of key concepts(15,16). This, combined with evidence of poor numeracy skills(17), may make it difficult to critically interpret and utilise evidence from RCTs. The culture and attitudes of clinicians are also highly relevant; in the case of surgery, there is evidence that surgeons tend to value clinical experience and individualism over research findings(18) and tend to resist changing established patterns of practice(19).

Proposed novel approach

Important insights into how clinicians and academics think about RCTs may be gained by examining the perspectives and opinions they convey when writing about RCTs in the published literature. Analysis of these views could improve understanding of the factors and issues of concern to clinicians when interpreting the results of an RCT and relating them to their own practice. The results may have importance across many areas, from trial design and reporting, to surgeon education, and organisational implications for surgical practice to promote uptake of effective new treatments.

This study protocol describes a new approach with the aim of establishing feasibility. It will explore the understanding, contextualisation and use of evidence from RCTs by examining existing literature that cite selected index RCTs. Surgical trials will be selected as case studies to pilot the method, to investigate its ability to capture relevant factors and potential challenges in implementation of trial findings associated with complex interventions. The results will inform progression to a larger-scale project to build upon preliminary findings.

For peer review only - http://bmjopen.bmj.com/site/about/guidelines.xhtml

METHODS AND ANALYSIS

Aim

This study aims to establish the feasibility of using a new method to generate insights into how evidence from RCTs is understood and assessed for potential application to clinical practice.

Objectives

This feasibility study has several objectives: to understand how evidence from surgical RCTs is used, understood and interpreted in published articles; to assess the methodological quality of the selected RCTs; to consider the quality of the trials alongside writings about the trials to generate insights into how evidence from RCTs may, or may not, be appropriately understood and utilised .

Design

The sequential steps involved in this study are summarised in table 1 and are described in the following sections.

1. *Purposefully select major surgical RCTs reported in the last ten years*

The parameters to be considered for purposeful sampling include: i) clinical area; ii) impact of the paper (journal bibliography and citation analysis); iii) type of intervention and comparator (e.g a novel invasive surgical procedure); iv) trial design (e.g. pragmatic or explanatory); v) country of trial conduct (single country or international). The index trials will be identified from a short-list of 20 top

For peer review only - http://bmjopen.bmj.com/site/about/guidelines.xhtml

cited major surgical RCTs published between 2006 and 2016 in English. RCTs will be identified in the Web of Science™ and Scopus® search engines. These tools combine primary search functionality with citation tracking to count and identify articles citing selected RCTs. In the first instance, Scopus will be searched using the following terms within the title field: (randomi*ed trial) AND (surg* or operat* or laparoscop* or *ectomy or *otomy or *plasty or *rrhaphy or *ostomy or *pexy or fusion or arthrodesis or arthroscop* or ((internal or external) and fixation) or (caesarean section) or (bypass and (artery or graft)) or ((repair or replace*) AND (hernia or aneurysm or valve or fistula or hip or knee or ankle or shoulder or elbow)) or ((resection or excision or dissection) AND (axillary or anterior or rect* or liver* or hepatic or gastric or colon* or colorectal* or (lymph node*) or (small bowel)))) AND NOT (chemotherap*). Search results will be sorted in descending numerical order of total citation count. Article titles and abstracts will be screened to identify the 20 most highly cited surgical RCTs (in descending order of number of citations from the Scopus database), involving 2 or more groups, with comparison between at least one surgical technique and another invasive treatment. The search will be repeated using the Web of Science search engine, and a consolidated list created. The short-list will be reviewed by all authors to decide which RCTs will be examined. The full text of the selected trial reports will be retrieved. Based on pilot work, future larger-scale application of this method may permit sampling of many RCTs across the entire range of criteria described above.

2. Identify and systematically sample the articles citing the selected RCTs

Articles citing each trial will be identified in two ways: using Web of Science™ and Scopus® citation tracking; and using the Altmetric 'bookmarklet' to identify social media and web-based articles(21).

The Web of Science™ and Scopus® search engines provide a list of all publications citing an individual article. There are 41 different types of document indexed by Web of Science, and 15 types indexed

For peer review only - http://bmjopen.bmj.com/site/about/guidelines.xhtml

by Scopus (see supplementary material table 1). From the list of articles citing the selected RCTs, the following types will be included from the Web of Science: discussion, editorial material and letter. The following documents will be selected from Scopus: editorial and letter. All other article types will be excluded. These types have been chosen because the authors consider that the selected document types are likely to contain a higher proportion of author opinions than other data sources. If initial searches yield insufficient data, this aspect of the method will be reviewed by the project team to widen the search strategy and include a larger sample of relevant articles. All citing articles of the selected types will be analysed. This will include articles that respond directly to the index RCTs, for example commenting on its design or findings, as well as those that cite the trials as part of broader or apparently unrelated discussions. This approach will help investigate the understanding and utilisation of evidence from RCTs in the broad setting of evidence-based medicine.

Further online articles citing the included RCTs will be identified using Altmetric. Altmetric is an alternative to conventional citation-based metrics that specifically examines online attention given to academic material. It includes citations on Wikipedia and in policy documents, as well as discussions on blogs, mainstream media and social networks such as Twitter. Combination with traditional citations in the indexed scientific literature will help create a broad picture of the articles citing surgical RCTs.

3. Undertake in-depth qualitative analysis and identify emerging themes

Eligible articles in both the traditional academic press and the online environment will be compiled in electronic format. Thematic analysis will be performed, using an inductive approach, to identify stated and latent themes and create a rich description of the data(22).

An initial sample of 5 articles will be carefully read and re-read in detail by BEB - a post-doctoral surgeon-researcher who has previous experience of qualitative research. Individual sections of text

For peer review only - http://bmjopen.bmj.com/site/about/guidelines.xhtml
For peer review only

will be coded to summarise content and meaning. As the project has very specific objectives, some of the likely codes/themes can be anticipated, but no *a priori* coding criteria will be specified, and all content of articles will be coded (albeit in more/less depth, in accordance with the project objectives). This will ensure that findings will be grounded in the data as much as possible. Coded text will be grouped into themes, creating hierarchies of super- and sub-ordinate nodes. The initial 5 articles will be independently double-coded by LR, a non-clinical lecturer in qualitative health sciences. BEB and LR will meet to discuss and review the initial five coded articles and seek to resolve any discrepancies in coding. Double-coding will be performed on a further 5 articles if there are "significant" discrepancies (which will be judged at the time of analysis but is likely to constitute discrepancies that extend beyond differences in how codes are named). The second round of double-coding, if conducted, will be followed by another review between coders. Thereafter, provided there is satisfactory agreement, all further analysis will be conducted independently by BEB. An iterative approach will continue, with further analysis until thematic saturation is achieved, with no new themes identified in 5 articles (or until the project ends).

After reaching saturation, the emergent themes will be discussed, iterated and refined with the wider research group, consisting of a senior trials methodologist and surgeon (JMB), and a senior medical anthropologist (HL). This will help create a coherent thematic structure that is meaningful to all stakeholders. In the final analysis, emergent themes will be organised and categorised to best explain the observed findings. All analysis will be conducted using NVivo for Windows 11.4 (QSR International Pty Ltd, Australia).

4. Summarise validity and reporting of included RCTs

After completing the qualitative analysis, the selected RCTs will be appraised using established, validated tools designed to assess their reporting, internal and external validity. This will involve

For peer review only - http://bmjopen.bmj.com/site/about/guidelines.xhtml

collection of data using the following tools: Consolidated Standards of Reporting Trials for Non-Pharmacological Treatments (CONSORT-NPT) checklist(23); Cochrane Risk of Bias Tool(9); Pragmatic-Explanatory Continuum Indicator Summary 2 (PRECIS-2) tool(20); Context and Implementation of Complex Interventions (CICI) checklist(24). Where necessary, protocol papers or documents will be sought to collect a complete dataset if possible. Simple study characteristics and publication information will also be compiled.

5. *Combine results to develop deeper understanding of how trials are understood, and the relationship with trial quality*

The results of the thematic analysis described in step 3 above will be compared with the included RCTs' methodological and reporting quality, as determined in step 4 described above. The results of both analyses will be collated to explore perceptions and understanding of authors that use and cite RCT evidence in relation to the methodological and reporting quality of the relevant index RCT.

6. *Develop and refine the methodology*

Pilot work will be conducted to establish the feasibility of using this new method. It is anticipated that the experience gained will result in iterative improvements to the method before future, larger scale application to investigate published reactions to other RCTs in surgery or in other areas of health care. For example, a framework may be produced for use in further reviews to categorise data emerging from the qualitative analysis. Future developments are likely to include purposive sampling of multiple RCTs using a range of different criteria to identify common themes and differences across different medical fields. It may also be useful to include a greater range of citing articles, such as other trials and review articles, rather than restricting the qualitative analysis to editorial, discussion and letter article types.

For peer review only - http://bmjopen.bmj.com/site/about/guidelines.xhtml

7. *Create recommendations to inform future design, conduct, reporting and implementation of surgical RCTs*

The findings of this study will lead to recommendations for future trial design and reporting to anticipate and avoid common criticisms and misunderstandings. Beyond RCT design, this study may also yield insights to optimise translation of evidence from high-quality RCTs into day-to-day clinical practiceby helping identify common perceptual barriers to the uptake of research evidence. For example, study findings may indicate a need for focused education for surgeons to improve understanding of when and how to implement new interventions based on RCT findings. This may improve efficiency in the generation and application of evidence on the clinical utility of surgical interventions, to the benefit of patients, clinicians and the health care system.

Ethical considerations

This study involves secondary use of written material that is already available in the public domain and does not require ethical review.

Patient and public involvement

Patients and members of the public were not involved in any aspect of the design of this study.

For peer review only - http://bmjopen.bmj.com/site/about/guidelines.xhtml

For peer review only

DISCUSSION

This protocol paper describes a new means of understanding how clinicians and academics respond to, and utilise, published trial data. The findings will be disseminated through conference presentation and peer-reviewed publication, as well as local social media channels. If successful, the results of pilot work will be used to inform a future, larger scale application of the method. This method has the potential to generate new knowledge both to improve RCT design and reporting, and to optimise translation of knowledge from RCTs into everyday clinical practice. In the future, this method may be complemented by other research techniques such as in-depth interviews, to build upon the findings and explore the issues identified.

For peer review only

For peer review only - http://bmjopen.bmj.com/site/about/guidelines.xhtml

Contributions

BEB and JMB conceived the study. BEB drafted the protocol. BEB, LR, HL and JMB provided intellectual input into improving the study design and revising the protocol. All authors have contributed to and approved the final manuscript.

Funding

B E Byrne is supported by the National Institute for Health Research. Jane Blazeby is a NIHR Senior Investigator. This work was undertaken with the support of the MRC ConDuCT-II (Collaboration and innovation for Difficult and Complex randomised controlled Trials In Invasive procedures) Hub for Trials Methodology Research (MR/K025643/1) and support from the Royal College of Surgeons of England Bristol Surgical Trials Centre. The views expressed are those of the authors and not necessarily those of the NHS, the NIHR or the Department of Health. The funders had no role in developing the protocol.

Competing interests statement

None declared.

For peer review only - http://bmjopen.bmj.com/site/about/guidelines.xhtml
For peer review only

References

1. Evidence-based Medicine Working Group. Evidence-based medicine: a new approach to teaching the practice of medicine. J Am Med Assoc [Internet]. 1992;268(17):2420–5. Available from: http://content.wkhealth.com/linkback/openurl?sid=WKPTLP:landingpage&an=00006534-201412000-00040
2. Oxford Centre for Evidence-Based Medicine. Levels of evidence [Internet]. 2009 [cited 2016 Jun 10]. Available from: http://www.cebm.net/oxford-centre-evidence-based-medicine-levels-evidence-march-2009/
3. Schuster MA, McGlynn EA, Brook RH. How good is the quality of health care in the United States? Milbank Q [Internet]. 1998;76(4):517–63. Available from: http://www.pubmedcentral.nih.gov/articlerender.fcgi?artid=2751100&tool=pmcentrez&rendertype=abstract
4. Freemantle N, Nazareth I, Eccles M, Wood J, Haines A. A randomised controlled trial of the effect of educational outreach by community pharmacists on prescribing in UK general practice. Br J Gen Pract. 2002;52(477):290–5.
5. Marzluf BA, Reichardt B, Neuhofer LM, Kogler B, Wolzt M. Influence of drug adherence and medical care on heart failure outcome in the primary care setting in Austria. Pharmacoepidemiol Drug Saf. 2015;24:722–30.
6. Arriaga AF, Lancaster RT, Berry WR, Regenbogen SE, Lipsitz SR, Kaafarani HMA, et al. The Better Colectomy Project: association of evidence-based best-practice adherence rates to outcomes in colorectal surgery. Ann Surg [Internet]. 2009 Oct [cited 2012 Oct 24];250(4):507–13. Available from: http://www.ncbi.nlm.nih.gov/pubmed/19734778
7. Deerenberg EB, Harlaar JJ, Steyerberg EW, Lont HE, van Doorn HC, Heisterkamp J, et al. Small bites versus large bites for closure of abdominal midline incisions (STITCH): a double-blind, multicentre, randomised controlled trial. Lancet. 2015;386(10000):1254–60.
8. Leaper D, Wilson P, Assadian O, Edmiston C, Kiernan M, Miller A, et al. The role of antimicrobial sutures in preventing surgical site infection. Ann R Coll Surg Engl. 2017;99(6):439–43.
9. Higgins JPT, Altman DG, Gøtzsche PC, Jüni P, Moher D, Oxman AD, et al. The Cochrane Collaboration's tool for assessing risk of bias in randomised trials. Br Med J [Internet]. 2011;343:d5928. Available from: http://www.bmj.com/cgi/doi/10.1136/bmj.d5928
10. Ahmad N, Boutron I, Moher D, Pitrou I, Roy C, Ravaud P. Neglected external validity in reports

For peer review only - http://bmjopen.bmj.com/site/about/guidelines.xhtml

of randomized trials: the example of hip and knee osteoarthritis. Arthritis Care Res. 2009;61(3):361–9.

11. Rothwell PM. External validity of randomised controlled trials: “To whom do the results of this trial apply?” Lancet. 2005;365:82–93.

12. Moore G, Audrey S, Barker M, Bond L, Bonnell C, Hardeman W, et al. Process evaluation of complex interventions: UK Medical Research Council (MRC) guidance [Internet]. 2015 [cited 2016 Jun 7]. Available from: https://www.mrc.ac.uk/documents/pdf/mrc-phsrn-process-evaluation-guidance-final/

13. Craig P, Dieppe P, Macintyre S, Michie S, Nazareth I, Petticrew M. Developing and evaluating complex interventions: The new Medical Research Council guidance. Int J Nurs Stud [Internet]. 2013;50(5):587–92. Available from: http://dx.doi.org/10.1016/j.ijnurstu.2012.09.010

14. Bate P, Robert G, Fulop N, Øvretveit J, Dixon-Woods M. Perspectives on context. Perspectives on Context. Health Foundation; 2014. 1-108 p.

15. Ziebland S, Featherstone K, Snowdon C, Barker K, Frost H, Fairbank J. Does it matter if clinicians recruiting for a trial don’t understand what the trial is really about? Qualitative study of surgeons’ experiences of participation in a pragmatic multi-centre RCT. Trials [Internet]. 2007;8:4. Available from: http://www.pubmedcentral.nih.gov/articlerender.fcgi?artid=1794540&tool=pmcentrez&rendertype=abstract

16. Potter S, Mills N, Cawthorn SJ, Donovan J, Blazeby JM. Time to be brave: is educating surgeons the key to unlocking the potential of randomised clinical trials in surgery? A qualitative study. Trials [Internet]. 2014;15:80. Available from: http://www.ncbi.nlm.nih.gov/pubmed/24628821

17. Garcia-Retamero R, Cokely ET, Wicki B, Joeris A. Improving risk literacy in surgeons. Patient Educ Couns [Internet]. 2016;99(7):1156–61. Available from: http://dx.doi.org/10.1016/j.pec.2016.01.013

18. Orri M, Farges O, Clavien P-A, Barkun J, Revah-Lévy A. Being a surgeon - the myth and the reality. A meta-synthesis of surgeons’ perspectives about factors affecting their practice and well-being. Ann Surg [Internet]. 2014;260(5):721–9. Available from: http://content.wkhealth.com/linkback/openurl?sid=WKPTLP:landingpage&an=00000658-201411000-00002

19. Kitto S, Petrovic A, Gruen RL, Smith JA. Evidence-based medicine training and implementation in surgery: the role of surgical cultures. J Eval Clin Pract. 2011;17:819–26.

For peer review only - http://bmjopen.bmj.com/site/about/guidelines.xhtml

For peer review only

20. Loudon K, Treweek S, Sullivan F, Donnan P, Thorpe KE, Zwarenstein M. The PRECIS-2 tool: designing trials that are fit for purpose. Br Med J [Internet]. 2015;350:h2147. Available from: http://www.bmj.com/cgi/doi/10.1136/bmj.h2147

21. Altmetric. Bookmarklet - Altmetric [Internet]. [cited 2017 Mar 21]. Available from: https://www.altmetric.com/products/free-tools/bookmarklet/

22. Braun V, Clarke V. Using thematic analysis in psychology. Qual Res Psychol [Internet]. 2006 Jan [cited 2014 May 25];3(2):77–101. Available from: http://www.tandfonline.com/doi/abs/10.1191/1478088706qp063oa

23. Boutron I, Moher D, Altman DG, Schulz KF, Ravaud P. Methods and processes of the CONSORT Group: example of an extension for trials assessing nonpharmacologic treatments. Ann Intern Med [Internet]. 2008;148:295–309. Available from: http://dx.doi.org/10.7326/0003-4819-148-4-200802190-00008

24. Pfadenhauer L, Rohwer A, Burns J, Booth A, Lysdahl KB, Hofmann B, et al. Guidance for the assessment of context and implementation in Health Technology Assessments (HTA) and systematic reviews of complex interventions: the Context and Implementation of Complex Interventions (CICI) framework [Internet]. 2016 [cited 2016 Mar 8]. Available from: http://www.intergrate-hta.eu/downloads

For peer review only

For peer review only - http://bmjopen.bmj.com/site/about/guidelines.xhtml

TABLES

Table 1. Method outline for identifying and analysing articles citing surgical RCTs.

Step	Action
1	Purposefully select major surgical RCTs reported in the last ten years.
2	Identify and systematically sample the articles citing the selected RCTs.
3	Undertake in-depth qualitative analysis and identify emerging themes.
4	Summarise validity and reporting of included RCTs.
5	Combine the results of steps 3 and 4 to develop deeper understanding of how trials are understood, and the relationship with trial quality.
6	Develop and refine the methodology.
7	Create recommendations to inform future design, conduct, reporting and implementation of surgical RCTs.

For peer review only

For peer review only - http://bmjopen.bmj.com/site/about/guidelines.xhtml

SUPPLEMENTARY MATERIAL

Supplementary table 1. Document types indexed by Web of Science and Scopus.

Web of Science	Scopus
Article	Article
Abstract of published item	Abstract report
Art exhibit review	Article in press
Bibliography	Book
Biographical-item	Business article
Book	Book chapter
Book chapter	Conference paper
Book review	Conference review
Chronology	Editorial
Correction	Erratum
Correction, addition	Letter
Dance performance review	Note
Data paper	Press release
Database review	Review
Discussion	Short survey
Editorial material	
Excerpt	
Fiction, creative prose	
Film review	
Hardware review	
Item about an individual	
Letter	
Meeting abstract	
Meeting summary	
Music performance review	
Music score	
Music score review	
News item	
Note	
Poetry	
Proceedings paper	
Record review	
Reprint	
Retracted publication	
Retraction	
Review	
Script	
Software review	
TV review, radio review	
TV review, radio review video	
Theatre review	

For peer review only

For peer review only - http://bmjopen.bmj.com/site/about/guidelines.xhtml

BMJ Open

Evidence into practice: protocol for a new mixed methods approach to explore the relationship between trials evidence and clinical practice through systematic identification and analysis of articles citing randomised controlled trials.

Journal:	*BMJ Open*
Manuscript ID	bmjopen-2018-023215.R1
Article Type:	Protocol
Date Submitted by the Author:	20-Jul-2018
Complete List of Authors:	Byrne, Benjamin; University of Bristol, Centre for Surgical Research, Population Health Sciences<br>Rooshenas, Leila; University of Bristol, Centre for Surgical Research, Population Health Sciences<br>Lambert, Helen; University of Bristol, Population Health Sciences<br>Blazeby, Jane; University of Bristol, Centre for Surgical Research, Population Health Sciences
**Primary Subject Heading**:	Evidence based practice
Secondary Subject Heading:	Health services research, Qualitative research, Research methods, Surgery
Keywords:	QUALITATIVE RESEARCH, STATISTICS & RESEARCH METHODS, SURGERY, Clinical trials < THERAPEUTICS

Evidence into practice: protocol for a new mixed methods approach to explore the relationship between trials evidence and clinical practice through systematic identification and analysis of articles citing randomised controlled trials.

B E Byrne[1], L Rooshenas[1], H Lambert[2], J M Blazeby[1]

[1]Centre for Surgical Research, Population Health Sciences, Bristol Medical School, University of Bristol, Bristol, UK.

[2]Population Health Sciences, Bristol Medical School, University of Bristol, Bristol, UK.

Correspondence: B E Byrne

Address: Centre for Surgical Research, Population Health Sciences, Bristol Medical School, University of Bristol, Canynge Hall, 39 Whatley Road, Clifton, Bristol BS8 2PS, UK.

Email: benbyrne@doctors.org.uk.

Telephone: 0117 928 7279

For peer review only - http://bmjopen.bmj.com/site/about/guidelines.xhtml

For peer review only

ABSTRACT

Introduction: Randomised controlled trials (RCTs) provide high-quality evidence to inform practice. However, much routine care is not based upon available RCT evidence. Understanding this disconnect may improve trial design, reporting and implementation. Published literature commenting on RCTs may yield relevant insights. This protocol presents a new approach examining how researchers understand, contextualise and use evidence from RCTs, through analysis of letters, editorials and discussion pieces citing individual RCTs. Surgical case studies will illustrate its ability to identify wide ranging factors influencing application of trials evidence.

Methods and analysis: In-depth study of published literature will explore written responses to RCTs. After purposefully selecting individual RCTs, Web of Science and Scopus searches will systematically identify all citing articles. Editorials, discussions and letters will be included. These are considered most likely to provide critiques and opinions about the index RCTs. Original articles and reviews will be excluded. Clinical specialty, RCT design, outcomes and bibliographic data will be collected for the RCTs and citing articles. Citing articles will be thematically analysed using the constant comparison technique to explore author understanding, contextualisation and relationship to clinical practice for the index trial. Coding will include generic issues relevant to all RCTs, such as sample size or blinding, and features specific to surgery, such as learning curve. Index trial quality will be examined using validated tools. Results will be combined to create a broad overview of the understanding and use of RCT evidence.

Ethics and dissemination: This study involves secondary use of existing articles and does not require ethical approval. Pilot work will establish its feasibility and inform progression to larger-scale utilisation across a broad range of RCTs. Findings will be published in a peer reviewed journal and

For peer review only - http://bmjopen.bmj.com/site/about/guidelines.xhtml

presented at surgical and methodological conferences. Results will guide future work on trial design to optimise implementation of results.

For peer review only

For peer review only - http://bmjopen.bmj.com/site/about/guidelines.xhtml

ARTICLE SUMMARY

Strengths and limitations of this study

- This new method will use published response to RCTs to identify how clinicians and academics interpret and use evidence from randomised trials.
- The emerging data will identify areas that need attention to improve translation of RCT results into practice.
- Rigorous qualitative analysis combined with structured data extraction on the quality and reporting of included RCTs will be used to gain in-depth understanding of the relevant issues.
- This method may have broad applicability across all randomised trials.
- The study findings may be limited by the focus on included and published articles.

For peer review only

For peer review only - http://bmjopen.bmj.com/site/about/guidelines.xhtml

INTRODUCTION

Since the early description of evidence-based medicine[1], it has been recognised that high quality research is needed to improve clinical practice and patient outcomes. This typically includes well-designed and conducted randomised controlled trials (RCTs) and systematic reviews summarising the results of multiple trials in one field[2]. Such research is used to inform national guidelines which aim to influence and set standards for clinical practice.

Research across a number of areas, including surgery, has shown that day-to-day care is often not delivered in accordance with national guidelines and best evidence[3–6]. There are many reasons for lack of translation of knowledge from RCTs into practice. Trials may be compromised by problems with their design and conduct, limiting the credibility of the associated results and / or the applicability of the trial in other settings. Internal validity can be compromised by well-known sources of bias that may be assessed with validated tools, such as the Risk of Bias Tool[7]. Trial design and reporting may also compromise external validity, limiting the application of trial findings more broadly[8,9]. In addition, complex interventions, such as surgery, may be influenced by a wide range of factors, such as surgeon or institution caseload, teamwork, care pathways and organisational culture, which are generally referred to as 'context'[10–12]. The exact mechanisms by which such contextual elements could affect the results of care have not been defined, but they may include technical skills, decision-making, professional status that may influence quality of post-surgical care and communication. These may have clear relevance to both the efficacy and implementation of an intervention.

Other factors beyond the control or influence of trials methodologists are also important in determining whether evidence from RCTs is implemented in practice. Evidence from an individual RCT regarding a particular intervention must be considered alongside a range of other evidence

For peer review only - http://bmjopen.bmj.com/site/about/guidelines.xhtml
For peer review only

types, including RCTs concerning the same or similar interventions. Clinicians, many of whom have not been involved in clinical trials, may lack familiarity with trial design; even those involved in trial recruitment may have a poor grasp of key concepts[13,14]. This, combined with evidence of poor numeracy skills[15], may make it difficult to critically interpret and utilise evidence from RCTs. The culture and attitudes of clinicians are also highly relevant; in the case of surgery, there is evidence that surgeons tend to value clinical experience and individualism over research findings[16] and tend to resist changing established patterns of practice[17].

Proposed novel approach

Important insights into how clinicians and academics think about RCTs may be gained by examining the perspectives and opinions they convey when writing about RCTs in the published literature. Analysis of these views could improve understanding of the factors and issues of concern to clinicians when interpreting the results of an RCT and relating them to their own practice. The results may have importance across many areas, from trial design and reporting, to surgeon education, and organisational implications for surgical practice to promote uptake of effective new treatments.

This study protocol describes a new approach with the aim of establishing feasibility. It will explore the understanding, contextualisation and use of evidence from RCTs by examining existing literature that cite selected index RCTs. Surgical trials will be selected as case studies to pilot the method, to investigate its ability to capture relevant factors and potential challenges in implementation of trial findings associated with complex interventions. The results will inform progression to a larger-scale project to build upon preliminary findings.

For peer review only - http://bmjopen.bmj.com/site/about/guidelines.xhtml

METHODS AND ANALYSIS

Aim

This study aims to establish the feasibility of using a new method to generate insights into how evidence from RCTs is understood and assessed for potential application to clinical practice.

Objectives

This feasibility study has several objectives: to understand how evidence from surgical RCTs is used, understood and interpreted in published articles; to assess the methodological quality of the selected RCTs; to consider the quality of the trials alongside writings about the trials to generate insights into how evidence from RCTs may, or may not, be appropriately understood and utilised.

Design

The sequential steps involved in this study are summarised in table 1 and are described in the following sections.

1. *Purposefully select major surgical RCTs reported in the last ten years*

The parameters to be considered for purposeful sampling include: i) clinical area; ii) impact of the paper (journal impact and citation analysis); iii) type of intervention and comparator (e.g. a novel invasive surgical procedure); iv) trial design (e.g. pragmatic or explanatory); v) country of trial conduct (single country or international). The index trials will be identified from a short-list of 20 top

For peer review only - http://bmjopen.bmj.com/site/about/guidelines.xhtml

cited major surgical RCTs published between 2006 and 2016 in English. This timeframe will allow trials to accrue citations, while also focusing on evidence still relevant to current practice. RCTs will be identified in the Web of Science™ and Scopus® search engines. These tools combine primary search functionality with citation tracking to count and identify articles citing selected RCTs. In the first instance, Scopus will be searched using the following terms within the title field: (randomi*ed trial) AND (surg* or operat* or laparoscop* or *ectomy or *otomy or *plasty or *rrhaphy or *ostomy or *pexy or fusion or arthrodesis or arthroscop* or ((internal or external) and fixation) or (caesarean section) or (bypass and (artery or graft)) or ((repair or replace*) AND (hernia or aneurysm or valve or fistula or hip or knee or ankle or shoulder or elbow)) or ((resection or excision or dissection) AND (axillary or anterior or rect* or liver* or hepatic or gastric or colon* or colorectal* or (lymph node*) or (small bowel)))) AND NOT (chemotherap*). Search results will be sorted in descending numerical order of total citation count, as only a subset of citing articles will be included for analysis. Article titles and abstracts will be screened to identify the 20 most highly cited surgical RCTs (in descending order of number of citations from the Scopus database), involving 2 or more groups, with comparison between at least one surgical technique and another invasive treatment. The search will be repeated using the Web of Science search engine, and a consolidated list created. The short-list will be reviewed by all authors to decide which RCTs will be examined. The full text of the selected trial reports will be retrieved. Based on pilot work, future larger-scale application of this method may permit sampling of many RCTs across the entire range of criteria described above.

2. Identify and systematically sample the articles citing the selected RCTs

Articles citing each trial will be identified in two ways: using Web of Science™ and Scopus® citation tracking; and using the Altmetric.com ‘bookmarklet’ to identify social media and web-based articles[18].

For peer review only - http://bmjopen.bmj.com/site/about/guidelines.xhtml

The Web of Science™ and Scopus® search engines provide a list of all publications citing an individual article. There are 41 different types of document indexed by Web of Science, and 15 types indexed by Scopus (see supplementary material table 1). From the list of articles citing the selected RCTs, the following types will be included from the Web of Science: discussion, editorial material and letter. The following documents will be selected from Scopus: editorial and letter. All other article types will be excluded. These types have been chosen because the authors consider that the selected document types are likely to contain a higher proportion of author opinions than other data sources. If initial searches yield insufficient data, this aspect of the method will be reviewed by the project team to widen the search strategy and include a larger sample of relevant articles. All citing articles of the selected types will be analysed. This will include articles that respond directly to the index RCTs, for example commenting on its design or findings, as well as those that cite the trials as part of broader or apparently unrelated discussions. This approach will help investigate the understanding and utilisation of evidence from RCTs in the broad setting of evidence-based medicine.

Further online articles citing the included RCTs will be identified using Altmetric. Altmetric is an alternative to conventional citation-based metrics that specifically examines online attention given to academic material. It includes citations on Wikipedia and in policy documents, as well as discussions on blogs, mainstream media and social networks such as Twitter. Combination with traditional citations in the indexed scientific literature will help create a broad picture of the articles citing surgical RCTs.

3. Undertake in-depth qualitative analysis and identify emerging themes

Eligible articles in both the traditional academic press and the online environment will be compiled in electronic format. Given the focused objectives of this project, a formal grounded theory approach will not be adopted[19]. However, thematic analysis will be performed using the constant

For peer review only - http://bmjopen.bmj.com/site/about/guidelines.xhtml

comparison technique, adopted from grounded theory, to identify stated and latent themes and create a rich description of the data[20]. All analysis will be conducted using NVivo for Windows 11.4 (QSR International Pty Ltd, Australia).

An initial sample of 5 articles will be carefully read and re-read in detail by BEB - a post-doctoral surgeon-researcher who has previous experience of qualitative research. Individual sections of text will be coded to summarise content and meaning. As the project has very specific objectives, some of the likely codes/themes can be anticipated, but no *a priori* coding criteria will be specified, and all content of articles will be coded (albeit in more/less depth, in accordance with the project objectives). This will ensure that findings will be grounded in the data as much as possible. Coded text will be grouped into themes, creating hierarchies of super- and sub-ordinate nodes. The initial 5 articles will be independently double-coded by LR, a non-clinical lecturer in qualitative health sciences. BEB and LR will meet to discuss and review the initial five coded articles and seek to resolve any discrepancies in coding. Double-coding will be performed on a further 5 articles if there are "significant" discrepancies (which will be judged at the time of analysis but is likely to constitute discrepancies that extend beyond differences in how codes are named). The second round of double-coding, if conducted, will be followed by another review between coders. Thereafter, provided there is satisfactory agreement, further analysis will be conducted by BEB. An iterative approach will continue, with further analysis until thematic saturation is achieved, with no new themes identified in 5 articles (or until the project ends). An evolving descriptive account will be created, circulated and discussed at regular face-to-face meetings to revise and refine themes as they emerge. BEB will also make reflexive notes on the coding process. The study team includes a senior trials methodologist and surgeon (JMB) and a senior medical anthropologist (HL). The extensive experience in qualitiative research and surgical trials of this multidisciplinary team will help create a coherent thematic structure that is meaningful to all stakeholders. In the final analysis, emergent themes will be organised and categorised to best explain the observed findings.

For peer review only - http://bmjopen.bmj.com/site/about/guidelines.xhtml

4. Summarise validity and reporting of included RCTs

After completing the qualitative analysis, the selected RCTs will be appraised using established, validated tools designed to assess their reporting, internal and external validity. This will involve collection of data using the following tools: Consolidated Standards of Reporting Trials for Non-Pharmacological Treatments (CONSORT-NPT) checklist[21]; Cochrane Risk of Bias Tool[7]; Pragmatic-Explanatory Continuum Indicator Summary 2 (PRECIS-2) tool[22]; Context and Implementation of Complex Interventions (CICI) checklist[23]. Where necessary, protocol papers or documents will be sought to collect a complete dataset if possible. Simple study characteristics and publication information will also be compiled.

5. Combine results to develop deeper understanding of how trials are understood, and the relationship with trial quality

The results of the thematic analysis described in step 3 above will be compared with the included RCTs' methodological and reporting quality, as determined in step 4 described above. The results of both analyses will be collated to explore perceptions and understanding of authors that use and cite RCT evidence in relation to the methodological and reporting quality of the relevant index RCT.

6. Develop and refine the methodology

Pilot work will be conducted to establish the feasibility of using this new method. It is anticipated that the experience gained will result in iterative improvements to the method before future, larger scale application to investigate published reactions to other RCTs in surgery or in other areas of health care. For example, a framework may be produced for use in further reviews to categorise

For peer review only - http://bmjopen.bmj.com/site/about/guidelines.xhtml
For peer review only

data emerging from the qualitative analysis. Future developments are likely to include purposive sampling of multiple RCTs using a range of different criteria to identify common themes and differences across different medical fields. It may also be useful to include a greater range of citing articles, such as other trials and review articles, rather than restricting the qualitative analysis to editorial, discussion and letter article types.

7. *Create recommendations to inform future design, conduct, reporting and implementation of surgical RCTs*

The findings of this study will lead to recommendations for future trial design and reporting to anticipate and avoid common criticisms and misunderstandings. Beyond RCT design, this study may also yield insights to optimise translation of evidence from high-quality RCTs into day-to-day clinical practice by helping identify common perceptual barriers to the uptake of research evidence. For example, study findings may indicate a need for focused education for surgeons to improve understanding of when and how to implement new interventions based on RCT findings. This may improve efficiency in the generation and application of evidence on the clinical utility of surgical interventions, to the benefit of patients, clinicians and the health care system.

Patient and public involvement

Patients and members of the public were not involved in any aspect of the design of this study.

For peer review only - http://bmjopen.bmj.com/site/about/guidelines.xhtml
For peer review only

ETHICS AND DISSEMINATION

This study involves secondary use of written material that is already publicly available and does not require ethical review.

This protocol paper describes a new means of understanding how clinicians and academics respond to, and utilise, published trial data. The findings will be disseminated through conference presentation and peer-reviewed publication, as well as social media channels. If successful, the results of pilot work will be used to inform a future, larger scale application of the method which will also be published and presented, and promoted through social media.

This method has the potential to generate new knowledge both to improve RCT design and reporting, and to optimise translation of knowledge from RCTs into everyday clinical practice. In the future, it may be complemented by other research techniques such as in-depth interviews, to build upon the findings and explore the issues identified. Any developments in understanding will be used by the trial team and our network to help improve design and implementation of trials that will be hosted or supported by the team and the associated Bristol Clinical Trials and Evaluation Unit.

For peer review only

For peer review only - http://bmjopen.bmj.com/site/about/guidelines.xhtml

Contributions

BEB and JMB conceived the study. BEB drafted the protocol. BEB, LR, HL and JMB provided intellectual input into improving the study design and revising the protocol. All authors have contributed to and approved the final manuscript.

Acknowledgements

We would like to thank Cath Borwick, Information Specialist at the University of Bristol, for helping develop the search strategy and highlighting other resources for identifying responses to trials.

Funding

B E Byrne is supported by the National Institute for Health Research (NIHR). Jane Blazeby is a NIHR Senior Investigator. This work was supported by the NIHR Biomedical Research Centre at the University Hospitals Bristol NHS Foundation Trust and the University of Bristol. This work was undertaken with the support of the MRC ConDuCT-II (Collaboration and innovation for Difficult and Complex randomised controlled Trials In Invasive procedures) Hub for Trials Methodology Research (MR/K025643/1) and support from the Royal College of Surgeons of England Bristol Surgical Trials Centre. The views expressed are those of the authors and not necessarily those of the NHS, the NIHR or the Department of Health. The funders had no role in developing the protocol.

Competing interests statement

None declared.

For peer review only - http://bmjopen.bmj.com/site/about/guidelines.xhtml
For peer review only

References

1 Evidence-based Medicine Working Group. Evidence-based medicine: a new approach to teaching the practice of medicine. *J Am Med Assoc* 1992;**268**:2420–5. doi:10.1097/PRS.0000000000000719

2 Oxford Centre for Evidence-Based Medicine. Levels of evidence. 2009.http://www.cebm.net/oxford-centre-evidence-based-medicine-levels-evidence-march-2009/ (accessed 10 Jun 2016).

3 Schuster MA, McGlynn EA, Brook RH. How good is the quality of health care in the United States? *Milbank Q* 1998;**76**:517–63. doi:10.1111/j.1468-0009.2005.00403.x

4 Freemantle N, Nazareth I, Eccles M, *et al.* A randomised controlled trial of the effect of educational outreach by community pharmacists on prescribing in UK general practice. *Br J Gen Pract* 2002;**52**:290–5.

5 Marzluf BA, Reichardt B, Neuhofer LM, *et al.* Influence of drug adherence and medical care on heart failure outcome in the primary care setting in Austria. *Pharmacoepidemiol Drug Saf* 2015;**24**:722–30. doi:10.1002/pds

6 Arriaga AF, Lancaster RT, Berry WR, *et al.* The Better Colectomy Project: association of evidence-based best-practice adherence rates to outcomes in colorectal surgery. *Ann Surg* 2009;**250**:507–13. doi:10.1097/SLA.0b013e3181b672bc

7 Higgins JPT, Altman DG, Gøtzsche PC, *et al.* The Cochrane Collaboration's tool for assessing risk of bias in randomised trials. *Br Med J* 2011;**343**:d5928. doi:10.1136/bmj.d5928

8 Ahmad N, Boutron I, Moher D, *et al.* Neglected external validity in reports of randomized trials: the example of hip and knee osteoarthritis. *Arthritis Care Res* 2009;**61**:361–9. doi:10.1002/art.24279

9 Rothwell PM. External validity of randomised controlled trials: 'To whom do the results of this trial apply?' *Lancet* 2005;**365**:82–93.

10 Moore G, Audrey S, Barker M, *et al.* Process evaluation of complex interventions: UK Medical Research Council (MRC) guidance. 2015. doi:http://www.populationhealthsciences.org/MRC-PHSRN-Process-evaluation-guidance-final-2-.pdf

11 Craig P, Dieppe P, Macintyre S, *et al.* Developing and evaluating complex interventions: The new Medical Research Council guidance. *Int J Nurs Stud* 2013;**50**:587–92. doi:10.1016/j.ijnurstu.2012.09.010

12 Bate P, Robert G, Fulop N, *et al.* *Perspectives on context*. Health Foundation 2014.

13 Ziebland S, Featherstone K, Snowdon C, *et al.* Does it matter if clinicians recruiting for a trial

For peer review only

For peer review only - http://bmjopen.bmj.com/site/about/guidelines.xhtml

don't understand what the trial is really about? Qualitative study of surgeons' experiences of participation in a pragmatic multi-centre RCT. *Trials* 2007;**8**:4. doi:10.1186/1745-6215-8-4

14 Potter S, Mills N, Cawthorn SJ, *et al.* Time to be brave: is educating surgeons the key to unlocking the potential of randomised clinical trials in surgery? A qualitative study. *Trials* 2014;**15**:80. doi:10.1186/1745-6215-15-80

15 Garcia-Retamero R, Cokely ET, Wicki B, *et al.* Improving risk literacy in surgeons. *Patient Educ Couns* 2016;**99**:1156–61. doi:10.1016/j.pec.2016.01.013

16 Orri M, Farges O, Clavien P-A, *et al.* Being a surgeon - the myth and the reality. A meta-synthesis of surgeons' perspectives about factors affecting their practice and well-being. *Ann Surg* 2014;**260**:721–9. doi:10.1097/SLA.0000000000000962

17 Kitto S, Petrovic A, Gruen RL, *et al.* Evidence-based medicine training and implementation in surgery: the role of surgical cultures. *J Eval Clin Pract* 2011;**17**:819–26. doi:10.1111/j.1365-2753.2010.01526.x

18 Altmetric. Bookmarklet - Altmetric. https://www.altmetric.com/products/free-tools/bookmarklet/ (accessed 21 Mar 2017).

19 Glaser BG, Strauss AL. *The discovery of grounded theory: strategies for qualitative research*. Aldine Transaction 1967. doi:10.2307/2575405

20 Braun V, Clarke V. Using thematic analysis in psychology. *Qual Res Psychol* 2006;**3**:77–101. doi:10.1191/1478088706qp063oa

21 Boutron I, Moher D, Altman DG, *et al.* Methods and processes of the CONSORT Group: example of an extension for trials assessing nonpharmacologic treatments. *Ann Intern Med* 2008;**148**:295–309. doi:10.7326/0003-4819-148-4-200802190-00008

22 Loudon K, Treweek S, Sullivan F, *et al.* The PRECIS-2 tool: designing trials that are fit for purpose. *Br Med J* 2015;**350**:h2147. doi:10.1136/bmj.h2147

23 Pfadenhauer L, Rohwer A, Burns J, *et al.* Guidance for the assessment of context and implementation in Health Technology Assessments (HTA) and systematic reviews of complex interventions: the Context and Implementation of Complex Interventions (CICI) framework. 2016. http://www.intergrate-hta.eu/downloads (accessed 8 Mar 2016).

For peer review only

For peer review only - http://bmjopen.bmj.com/site/about/guidelines.xhtml

TABLES

Table 1. Method outline for identifying and analysing articles citing surgical RCTs.

Step	Action
1	Purposefully select major surgical RCTs reported in the last ten years.
2	Identify and systematically sample the articles citing the selected RCTs.
3	Undertake in-depth qualitative analysis and identify emerging themes.
4	Summarise validity and reporting of included RCTs.
5	Combine the results of steps 3 and 4 to develop deeper understanding of how trials are understood, and the relationship with trial quality.
6	Develop and refine the methodology.
7	Create recommendations to inform future design, conduct, reporting and implementation of surgical RCTs.

For peer review only

For peer review only - http://bmjopen.bmj.com/site/about/guidelines.xhtml

SUPPLEMENTARY MATERIAL

Supplementary table 1. Document types indexed by Web of Science and Scopus.

Web of Science	Scopus
Article	Article
Abstract of published item	Abstract report
Art exhibit review	Article in press
Bibliography	Book
Biographical-item	Business article
Book	Book chapter
Book chapter	Conference paper
Book review	Conference review
Chronology	Editorial
Correction	Erratum
Correction, addition	Letter
Dance performance review	Note
Data paper	Press release
Database review	Review
Discussion	Short survey
Editorial material	
Excerpt	
Fiction, creative prose	
Film review	
Hardware review	
Item about an individual	
Letter	
Meeting abstract	
Meeting summary	
Music performance review	
Music score	
Music score review	
News item	
Note	
Poetry	
Proceedings paper	
Record review	
Reprint	
Retracted publication	
Retraction	
Review	
Script	
Software review	
TV review, radio review	
TV review, radio review video	
Theatre review	

For peer review only - http://bmjopen.bmj.com/site/about/guidelines.xhtml
For peer review only

BMJ Open

Evidence into practice: protocol for a new mixed methods approach to explore the relationship between trials evidence and clinical practice through systematic identification and analysis of articles citing randomised controlled trials.

Journal:	*BMJ Open*
Manuscript ID	bmjopen-2018-023215.R2
Article Type:	Protocol
Date Submitted by the Author:	25-Sep-2018
Complete List of Authors:	Byrne, Benjamin; University of Bristol, Centre for Surgical Research, Population Health Sciences<br>Rooshenas, Leila; University of Bristol, Centre for Surgical Research, Population Health Sciences<br>Lambert, Helen; University of Bristol, Population Health Sciences<br>Blazeby, Jane; University of Bristol, Centre for Surgical Research, Population Health Sciences
**Primary Subject Heading**:	Evidence based practice
Secondary Subject Heading:	Health services research, Qualitative research, Research methods, Surgery
Keywords:	QUALITATIVE RESEARCH, STATISTICS & RESEARCH METHODS, SURGERY, Clinical trials < THERAPEUTICS

Evidence into practice: protocol for a new mixed methods approach to explore the relationship between trials evidence and clinical practice through systematic identification and analysis of articles citing randomised controlled trials.

B E Byrne[1], L Rooshenas[1], H Lambert[2], J M Blazeby[1]

[1]Centre for Surgical Research, Population Health Sciences, Bristol Medical School, University of Bristol, Bristol, UK.

[2]Population Health Sciences, Bristol Medical School, University of Bristol, Bristol, UK.

Correspondence: B E Byrne

Address: Centre for Surgical Research, Population Health Sciences, Bristol Medical School, University of Bristol, Canynge Hall, 39 Whatley Road, Clifton, Bristol BS8 2PS, UK.

Email: benbyrne@doctors.org.uk.

Telephone: 0117 928 7279

For peer review only

For peer review only - http://bmjopen.bmj.com/site/about/guidelines.xhtml

ABSTRACT

Introduction: Randomised controlled trials (RCTs) provide high-quality evidence to inform practice. However, much routine care is not based upon available RCT evidence. Understanding this disconnect may improve trial design, reporting and implementation. Published literature commenting on RCTs may yield relevant insights. This protocol presents a new approach examining how researchers understand, contextualise and use evidence from RCTs, through analysis of letters, editorials and discussion pieces citing individual RCTs. Surgical case studies will illustrate its ability to identify wide ranging factors influencing application of trials evidence.

Methods and analysis: In-depth study of published literature will explore written responses to RCTs. After purposefully selecting individual RCTs, we will systematically identify all citing articles covered in Web of Science and Scopus. Editorials, discussions and letters will be included. These are considered most likely to provide critiques and opinions about index RCTs. Original articles and reviews will be excluded. Clinical specialty, RCT design, outcomes and bibliographic data will be collected for RCTs and citing articles. Citing articles will be thematically analysed using the constant comparison technique to explore author understanding, contextualisation and relationship to clinical practice for the index trial. Coding will include generic issues relevant to all RCTs, such as sample size or blinding, and features specific to surgery, such as learning curve. Index trial quality will be examined using validated tools. Results will be combined to create a broad overview of the understanding and use of RCT evidence.

Ethics and dissemination: This study involves secondary use of existing articles and does not require ethical approval. Pilot work will establish its feasibility and inform progression to larger-scale utilisation across a broad range of RCTs. Findings will be published in a peer reviewed journal and

For peer review only - http://bmjopen.bmj.com/site/about/guidelines.xhtml
For peer review only

presented at surgical and methodological conferences. Results will guide future work on trial design to optimise implementation of results.

For peer review only

For peer review only - http://bmjopen.bmj.com/site/about/guidelines.xhtml

ARTICLE SUMMARY

Strengths and limitations of this study

- This new method will use published response to RCTs to identify how clinicians and academics interpret and use evidence from randomised trials.
- The emerging data will identify areas that need attention to improve translation of RCT results into practice.
- Rigorous qualitative analysis combined with structured data extraction on the quality and reporting of included RCTs will be used to gain in-depth understanding of the relevant issues.
- This method may have broad applicability across all randomised trials.
- The study findings may be limited by the focus on included and published articles.

For peer review only - http://bmjopen.bmj.com/site/about/guidelines.xhtml

INTRODUCTION

Since the early description of evidence-based medicine[1], it has been recognised that high quality research is needed to improve clinical practice and patient outcomes. This typically includes well-designed and conducted randomised controlled trials (RCTs) and systematic reviews summarising the results of multiple trials in one field[2]. Such research is used to inform national guidelines which aim to influence and set standards for clinical practice.

Research across a number of areas, including surgery, has shown that day-to-day care is often not delivered in accordance with national guidelines and best evidence[3–6]. There are many reasons for lack of translation of knowledge from RCTs into practice. Trials may be compromised by problems with their design and conduct, limiting the credibility of the associated results and / or the applicability of the trial in other settings. Internal validity can be compromised by well-known sources of bias that may be assessed with validated tools, such as the Risk of Bias Tool[7]. Trial design and reporting may also compromise external validity, limiting the application of trial findings more broadly[8,9]. In addition, complex interventions, such as surgery, may be influenced by a wide range of factors, such as surgeon or institution caseload, teamwork, care pathways and organisational culture, which are generally referred to as 'context'[10–12]. The exact mechanisms by which such contextual elements could affect the results of care have not been defined, but they may include technical skills, decision-making, professional status that may influence quality of post-surgical care and communication. These may have clear relevance to both the efficacy and implementation of an intervention.

Other factors beyond the control or influence of trials methodologists are also important in determining whether evidence from RCTs is implemented in practice. Evidence from an individual RCT regarding a particular intervention must be considered alongside a range of other evidence

For peer review only - http://bmjopen.bmj.com/site/about/guidelines.xhtml

types, including RCTs concerning the same or similar interventions. Clinicians, many of whom have not been involved in clinical trials, may lack familiarity with trial design; even those involved in trial recruitment may have a poor grasp of key concepts[13,14]. This, combined with evidence of poor numeracy skills[15], may make it difficult to critically interpret and utilise evidence from RCTs. The culture and attitudes of clinicians are also highly relevant; in the case of surgery, there is evidence that surgeons tend to value clinical experience and individualism over research findings[16] and tend to resist changing established patterns of practice[17].

Proposed novel approach

Important insights into how clinicians and academics think about RCTs may be gained by examining the perspectives and opinions they convey when writing about RCTs in the published literature. Analysis of these views could improve understanding of the factors and issues of concern to clinicians when interpreting the results of an RCT and relating them to their own practice. The results may have importance across many areas, from trial design and reporting, to surgeon education, and organisational implications for surgical practice to promote uptake of effective new treatments.

This study protocol describes a new approach with the aim of establishing feasibility. It will explore the understanding, contextualisation and use of evidence from RCTs by examining existing literature that cite selected index RCTs. Surgical trials will be selected as case studies to pilot the method, to investigate its ability to capture relevant factors and potential challenges in implementation of trial findings associated with complex interventions. The results will inform progression to a larger-scale project to build upon preliminary findings.

For peer review only - http://bmjopen.bmj.com/site/about/guidelines.xhtml

METHODS AND ANALYSIS

Aim

This study aims to establish the feasibility of using a new method to generate insights into how evidence from RCTs is understood and assessed for potential application to clinical practice.

Objectives

This feasibility study has several objectives: to understand how evidence from surgical RCTs is used, understood and interpreted in published articles; to assess the methodological quality of the selected RCTs; to consider the quality of the trials alongside writings about the trials to generate insights into how evidence from RCTs may, or may not, be appropriately understood and utilised.

Design

The sequential steps involved in this study are summarised in table 1 and are described in the following sections.

1. *Purposefully select major surgical RCTs reported in the last ten years*

The parameters to be considered for purposeful sampling include: i) clinical area; ii) impact of the paper (journal impact and citation analysis); iii) type of intervention and comparator (e.g. a novel invasive surgical procedure); iv) trial design (e.g. pragmatic or explanatory); v) country of trial conduct (single country or international). The index trials will be identified from a short-list of 20 top

For peer review only - http://bmjopen.bmj.com/site/about/guidelines.xhtml

cited major surgical RCTs published between 2006 and 2016 in English. This timeframe will allow trials to accrue citations, while also focusing on evidence still relevant to current practice. RCTs will be identified in the Web of Science™ and Scopus® search engines. These tools combine primary search functionality with citation tracking to count and identify articles citing selected RCTs. In the first instance, Scopus will be searched using the following terms within the title field: (randomi*ed trial) AND (surg* or operat* or laparoscop* or *ectomy or *otomy or *plasty or *rrhaphy or *ostomy or *pexy or fusion or arthrodesis or arthroscop* or ((internal or external) and fixation) or (caesarean section) or (bypass and (artery or graft)) or ((repair or replace*) AND (hernia or aneurysm or valve or fistula or hip or knee or ankle or shoulder or elbow)) or ((resection or excision or dissection) AND (axillary or anterior or rect* or liver* or hepatic or gastric or colon* or colorectal* or (lymph node*) or (small bowel)))) AND NOT (chemotherap*). Search results will be sorted in descending numerical order of total citation count, as only a subset of citing articles will be included for analysis. Article titles and abstracts will be screened to identify the 20 most highly cited surgical RCTs (in descending order of number of citations from the Scopus database), involving 2 or more groups, with comparison between at least one surgical technique and another invasive treatment. The search will be repeated using the Web of Science search engine, and a consolidated list created. The short-list will be reviewed by all authors to decide which RCTs will be examined. The full text of the selected trial reports will be retrieved. Based on pilot work, future larger-scale application of this method may permit sampling of many RCTs across the entire range of criteria described above.

2. Identify and systematically sample the articles citing the selected RCTs

Articles citing each trial will be identified in two ways: using Web of Science™ and Scopus® citation tracking; and using the Altmetric.com 'bookmarklet' to identify social media and web-based articles[18].

For peer review only - http://bmjopen.bmj.com/site/about/guidelines.xhtml

The Web of Science™ and Scopus® search engines provide a list of all publications citing an individual article. There are 41 different types of document indexed by Web of Science, and 15 types indexed by Scopus (see supplementary material table 1). From the list of articles citing the selected RCTs, the following types will be included from the Web of Science: discussion, editorial material and letter. The following documents will be selected from Scopus: editorial and letter. All other article types will be excluded. These types have been chosen because the authors consider that the selected document types are likely to contain a higher proportion of author opinions than other data sources. If initial searches yield insufficient data, this aspect of the method will be reviewed by the project team to widen the search strategy and include a larger sample of relevant articles. All citing articles of the selected types will be analysed. This will include articles that respond directly to the index RCTs, for example commenting on its design or findings, as well as those that cite the trials as part of broader or apparently unrelated discussions. This approach will help investigate the understanding and utilisation of evidence from RCTs in the broad setting of evidence-based medicine.

Further online articles citing the included RCTs will be identified using Altmetric.com. Altmetric.com is one available source of alternative metrics, or ‘altmetrics’. Altmetrics are an alternative to conventional citation-based metrics that specifically examine online attention given to academic material[19]. They include citations on Wikipedia and in policy documents, as well as discussions on blogs, mainstream media and social networks such as Twitter. Combination with traditional citations in the indexed scientific literature will help create a broad picture of the articles citing surgical RCTs.

3. Undertake in-depth qualitative analysis and identify emerging themes

Eligible articles in both the traditional academic press and the online environment will be compiled in electronic format. Given the focused objectives of this project, a formal grounded theory approach will not be adopted[20]. However, thematic analysis will be performed using the constant

For peer review only - http://bmjopen.bmj.com/site/about/guidelines.xhtml

comparison technique, adopted from grounded theory, to identify stated and latent themes and create a rich description of the data[21]. All analysis will be conducted using NVivo for Windows 11.4 (QSR International Pty Ltd, Australia).

An initial sample of 5 articles will be carefully read and re-read in detail by BEB - a post-doctoral surgeon-researcher who has previous experience of qualitative research. Individual sections of text will be coded to summarise content and meaning. As the project has very specific objectives, some of the likely codes/themes can be anticipated, but no *a priori* coding criteria will be specified, and all content of articles will be coded (albeit in more/less depth, in accordance with the project objectives). This will ensure that findings will be grounded in the data as much as possible. Coded text will be grouped into themes, creating hierarchies of super- and sub-ordinate nodes. The initial 5 articles will be independently double-coded by LR, a non-clinical lecturer in qualitative health sciences. BEB and LR will meet to discuss and review the initial five coded articles and seek to resolve any discrepancies in coding. Double-coding will be performed on a further 5 articles if there are "significant" discrepancies (which will be judged at the time of analysis but is likely to constitute discrepancies that extend beyond differences in how codes are named). The second round of double-coding, if conducted, will be followed by another review between coders. Thereafter, provided there is satisfactory agreement, further analysis will be conducted by BEB. An iterative approach will continue, with further analysis until thematic saturation is achieved, with no new themes identified in 5 articles (or until the project ends). An evolving descriptive account will be created, circulated and discussed at regular face-to-face meetings to revise and refine themes as they emerge. BEB will also make reflexive notes on the coding process. The study team includes a senior trials methodologist and surgeon (JMB) and a senior medical anthropologist (HL). The extensive experience in qualitative research and surgical trials of this multidisciplinary team will help create a coherent thematic structure that is meaningful to all stakeholders. In the final analysis, emergent themes will be organised and categorised to best explain the observed findings.

For peer review only - http://bmjopen.bmj.com/site/about/guidelines.xhtml

4. Summarise validity and reporting of included RCTs

After completing the qualitative analysis, the selected RCTs will be appraised using established, validated tools designed to assess their reporting, internal and external validity. This will involve collection of data using the following tools: Consolidated Standards of Reporting Trials for Non-Pharmacological Treatments (CONSORT-NPT) checklist[22]; Cochrane Risk of Bias Tool[7]; Pragmatic-Explanatory Continuum Indicator Summary 2 (PRECIS-2) tool[23]; Context and Implementation of Complex Interventions (CICI) checklist[24]. Where necessary, protocol papers or documents will be sought to collect a complete dataset if possible. Simple study characteristics and publication information will also be compiled.

5. Combine results to develop deeper understanding of how trials are understood, and the relationship with trial quality

The results of the thematic analysis described in step 3 above will be compared with the included RCTs' methodological and reporting quality, as determined in step 4 described above. The results of both analyses will be collated to explore perceptions and understanding of authors that use and cite RCT evidence in relation to the methodological and reporting quality of the relevant index RCT.

6. Develop and refine the methodology

Pilot work will be conducted to establish the feasibility of using this new method. It is anticipated that the experience gained will result in iterative improvements to the method before future, larger scale application to investigate published reactions to other RCTs in surgery or in other areas of health care. For example, a framework may be produced for use in further reviews to categorise

For peer review only - http://bmjopen.bmj.com/site/about/guidelines.xhtml
For peer review only

data emerging from the qualitative analysis. Future developments are likely to include purposive sampling of multiple RCTs using a range of different criteria to identify common themes and differences across different medical fields. It may also be useful to include a greater range of citing articles, such as other trials and review articles, rather than restricting the qualitative analysis to editorial, discussion and letter article types.

7. *Create recommendations to inform future design, conduct, reporting and implementation of surgical RCTs*

The findings of this study will lead to recommendations for future trial design and reporting to anticipate and avoid common criticisms and misunderstandings. Beyond RCT design, this study may also yield insights to optimise translation of evidence from high-quality RCTs into day-to-day clinical practice by helping identify common perceptual barriers to the uptake of research evidence. For example, study findings may indicate a need for focused education for surgeons to improve understanding of when and how to implement new interventions based on RCT findings. This may improve efficiency in the generation and application of evidence on the clinical utility of surgical interventions, to the benefit of patients, clinicians and the health care system.

Patient and public involvement

Patients and members of the public were not involved in any aspect of the design of this study.

For peer review only

For peer review only - http://bmjopen.bmj.com/site/about/guidelines.xhtml

ETHICS AND DISSEMINATION

This study involves secondary use of written material that is already publicly available and does not require ethical review.

This protocol paper describes a new means of understanding how clinicians and academics respond to, and utilise, published trial data. The findings will be disseminated through conference presentation and peer-reviewed publication, as well as social media channels. If successful, the results of pilot work will be used to inform a future, larger scale application of the method which will also be published and presented, and promoted through social media.

This method has the potential to generate new knowledge both to improve RCT design and reporting, and to optimise translation of knowledge from RCTs into everyday clinical practice. In the future, it may be complemented by other research techniques such as in-depth interviews, to build upon the findings and explore the issues identified. Any developments in understanding will be used by the trial team and our network to help improve design and implementation of trials that will be hosted or supported by the team and the associated Bristol Clinical Trials and Evaluation Unit.

For peer review only

For peer review only - http://bmjopen.bmj.com/site/about/guidelines.xhtml

Contributions

BEB and JMB conceived the study. BEB drafted the protocol. BEB, LR, HL and JMB provided intellectual input into improving the study design and revising the protocol. All authors have contributed to and approved the final manuscript.

Acknowledgements

We would like to thank Cath Borwick, Information Specialist at the University of Bristol, for helping develop the search strategy and highlighting other resources for identifying responses to trials.

Funding

B E Byrne is supported by the National Institute for Health Research (NIHR). Jane Blazeby is a NIHR Senior Investigator. This work was supported by the NIHR Biomedical Research Centre at the University Hospitals Bristol NHS Foundation Trust and the University of Bristol. This work was undertaken with the support of the MRC ConDuCT-II (Collaboration and innovation for Difficult and Complex randomised controlled Trials In Invasive procedures) Hub for Trials Methodology Research (MR/K025643/1) and support from the Royal College of Surgeons of England Bristol Surgical Trials Centre. The views expressed are those of the authors and not necessarily those of the NHS, the NIHR or the Department of Health. The funders had no role in developing the protocol.

Competing interests statement

None declared.

For peer review only - http://bmjopen.bmj.com/site/about/guidelines.xhtml
For peer review only

References

1 Evidence-based Medicine Working Group. Evidence-based medicine: a new approach to teaching the practice of medicine. *J Am Med Assoc* 1992;**268**:2420–5. doi:10.1097/PRS.0000000000000719

2 Oxford Centre for Evidence-Based Medicine. Levels of evidence. 2009.http://www.cebm.net/oxford-centre-evidence-based-medicine-levels-evidence-march-2009/ (accessed 25 Sep 2018).

3 Schuster MA, McGlynn EA, Brook RH. How good is the quality of health care in the United States? *Milbank Q* 1998;**76**:517–63. doi:10.1111/j.1468-0009.2005.00403.x

4 Freemantle N, Nazareth I, Eccles M, *et al.* A randomised controlled trial of the effect of educational outreach by community pharmacists on prescribing in UK general practice. *Br J Gen Pract* 2002;**52**:290–5.

5 Marzluf BA, Reichardt B, Neuhofer LM, *et al.* Influence of drug adherence and medical care on heart failure outcome in the primary care setting in Austria. *Pharmacoepidemiol Drug Saf* 2015;**24**:722–30. doi:10.1002/pds

6 Arriaga AF, Lancaster RT, Berry WR, *et al.* The Better Colectomy Project: association of evidence-based best-practice adherence rates to outcomes in colorectal surgery. *Ann Surg* 2009;**250**:507–13. doi:10.1097/SLA.0b013e3181b672bc

7 Higgins JPT, Altman DG, Gøtzsche PC, *et al.* The Cochrane Collaboration's tool for assessing risk of bias in randomised trials. *Br Med J* 2011;**343**:d5928. doi:10.1136/bmj.d5928

8 Ahmad N, Boutron I, Moher D, *et al.* Neglected external validity in reports of randomized trials: the example of hip and knee osteoarthritis. *Arthritis Care Res* 2009;**61**:361–9. doi:10.1002/art.24279

9 Rothwell PM. External validity of randomised controlled trials: 'To whom do the results of this trial apply?' *Lancet* 2005;**365**:82–93. doi:10.1016/S0140-6736(04)17670-8

10 Moore G, Audrey S, Barker M, *et al.* Process evaluation of complex interventions: UK Medical Research Council (MRC) guidance. 2015. doi:http://www.populationhealthsciences.org/MRC-PHSRN-Process-evaluation-guidance-final-2-.pdf

11 Craig P, Dieppe P, Macintyre S, *et al.* Developing and evaluating complex interventions: The new Medical Research Council guidance. *Int J Nurs Stud* 2013;**50**:587–92. doi:10.1016/j.ijnurstu.2012.09.010

12 Bate P, Robert G, Fulop N, *et al.* *Perspectives on context*. Health Foundation 2014.

13 Ziebland S, Featherstone K, Snowdon C, *et al.* Does it matter if clinicians recruiting for a trial

For peer review only - http://bmjopen.bmj.com/site/about/guidelines.xhtml
For peer review only

don't understand what the trial is really about? Qualitative study of surgeons' experiences of participation in a pragmatic multi-centre RCT. *Trials* 2007;**8**:4. doi:10.1186/1745-6215-8-4

14 Potter S, Mills N, Cawthorn SJ, *et al.* Time to be brave: is educating surgeons the key to unlocking the potential of randomised clinical trials in surgery? A qualitative study. *Trials* 2014;**15**:80. doi:10.1186/1745-6215-15-80

15 Garcia-Retamero R, Cokely ET, Wicki B, *et al.* Improving risk literacy in surgeons. *Patient Educ Couns* 2016;**99**:1156–61. doi:10.1016/j.pec.2016.01.013

16 Orri M, Farges O, Clavien P-A, *et al.* Being a surgeon - the myth and the reality. A meta-synthesis of surgeons' perspectives about factors affecting their practice and well-being. *Ann Surg* 2014;**260**:721–9. doi:10.1097/SLA.0000000000000962

17 Kitto S, Petrovic A, Gruen RL, *et al.* Evidence-based medicine training and implementation in surgery: the role of surgical cultures. *J Eval Clin Pract* 2011;**17**:819–26. doi:10.1111/j.1365-2753.2010.01526.x

18 Altmetric. Bookmarklet - Altmetric. https://www.altmetric.com/products/free-tools/bookmarklet/ (accessed 25 Sep 2018).

19 Priem J, Taraborelli D, Groth P, *et al.* altmetrics: a manifesto. http://altmetrics.org/manifesto/ (accessed 25 Sep 2018).

20 Glaser BG, Strauss AL. *The discovery of grounded theory: strategies for qualitative research*. Aldine Transaction 1967. doi:10.2307/2575405

21 Braun V, Clarke V. Using thematic analysis in psychology. *Qual Res Psychol* 2006;**3**:77–101. doi:10.1191/1478088706qp063oa

22 Boutron I, Moher D, Altman DG, *et al.* Methods and processes of the CONSORT Group: example of an extension for trials assessing nonpharmacologic treatments. *Ann Intern Med* 2008;**148**:295–309. doi:10.7326/0003-4819-148-4-200802190-00008

23 Loudon K, Treweek S, Sullivan F, *et al.* The PRECIS-2 tool: designing trials that are fit for purpose. *Br Med J* 2015;**350**:h2147. doi:10.1136/bmj.h2147

24 Pfadenhauer L, Rohwer A, Burns J, *et al.* Guidance for the assessment of context and implementation in Health Technology Assessments (HTA) and systematic reviews of complex interventions: the Context and Implementation of Complex Interventions (CICI) framework. 2016. https://www.integrate-hta.eu/wp-content/uploads/2016/02/Guidance-for-the-Assessment-of-Context-and-Implementation-in-HTA-and-Systematic-Reviews-of-Complex-Interventions-The-Co.pdf (accessed 25 Sep 2018).

For peer review only

For peer review only - http://bmjopen.bmj.com/site/about/guidelines.xhtml

TABLES

Table 1. Method outline for identifying and analysing articles citing surgical RCTs.

Step	Action
1	Purposefully select major surgical RCTs reported in the last ten years.
2	Identify and systematically sample the articles citing the selected RCTs.
3	Undertake in-depth qualitative analysis and identify emerging themes.
4	Summarise validity and reporting of included RCTs.
5	Combine the results of steps 3 and 4 to develop deeper understanding of how trials are understood, and the relationship with trial quality.
6	Develop and refine the methodology.
7	Create recommendations to inform future design, conduct, reporting and implementation of surgical RCTs.

For peer review only

For peer review only - http://bmjopen.bmj.com/site/about/guidelines.xhtml

SUPPLEMENTARY MATERIAL

Supplementary table 1. Document types indexed by Web of Science and Scopus.

Web of Science	Scopus
Article	Article
Abstract of published item	Abstract report
Art exhibit review	Article in press
Bibliography	Book
Biographical-item	Business article
Book	Book chapter
Book chapter	Conference paper
Book review	Conference review
Chronology	Editorial
Correction	Erratum
Correction, addition	Letter
Dance performance review	Note
Data paper	Press release
Database review	Review
Discussion	Short survey
Editorial material	
Excerpt	
Fiction, creative prose	
Film review	
Hardware review	
Item about an individual	
Letter	
Meeting abstract	
Meeting summary	
Music performance review	
Music score	
Music score review	
News item	
Note	
Poetry	
Proceedings paper	
Record review	
Reprint	
Retracted publication	
Retraction	
Review	
Script	
Software review	
TV review, radio review	
TV review, radio review video	
Theatre review	

For peer review only

For peer review only - http://bmjopen.bmj.com/site/about/guidelines.xhtml
